# Clinical impact of follicular oncocytic (Hürthle cell) carcinoma in comparison with corresponding classical follicular thyroid carcinoma

**DOI:** 10.1007/s00259-020-04952-2

**Published:** 2020-07-18

**Authors:** Vera Wenter, Nathalie L. Albert, Marcus Unterrainer, Freba Ahmaddy, Harun Ilhan, Annamirl Jellinek, Thomas Knösel, Peter Bartenstein, Christine Spitzweg, Sebastian Lehner, Andrei Todica

**Affiliations:** 1 Department of Nuclear Medicine, University Hospital, LMU Munich, Marchioninistr. 15, 81377 Munich, Germany; 2 Comprehensive Cancer Center (CCC LMU) and Interdisciplinary Center for Thyroid Carcinoma (ISKUM), University Hospital, LMU Munich, Munich, Germany; 3 Institute of Pathology, Faculty of Medicine, University Hospital, LMU Munich, Munich, Germany; 4 Department of Internal Medicine IV, University Hospital, LMU Munich, Munich, Germany

**Keywords:** Hürthle cell carcinoma, Oncocytic/oxyphilic follicular carcinoma, Follicular thyroid cancer, Radioablation

## Abstract

**Purpose:**

There are controversial debates if patients with Hürthle cell carcinoma, also known as oxyphilic or oncocytic cell follicular thyroid carcinoma, have a poorer outcome. In this study, we systematically evaluated the clinical outcome in a large patient cohort following thyroidectomy and initial I-131 radioactive iodine therapy (RIT).

**Methods:**

We retrospectively evaluated a total of 378 patients with diagnosed oncocytic follicular Hürthle cell carcinoma (OFTC) (*N* = 126) or with classical follicular thyroid carcinoma (FTC) (*N* = 252). Patients received thyroidectomy and complementary I-131 RIT. Clinical data regarding basic demographic characteristics, tumor grade, persistent disease and recurrence during follow-up, and disease-free, disease-specific, and overall survival were collected during follow-up of 6.9 years (interquartile range 3.7; 11.7 years). Univariate and multivariate analyses were used to identify factors associated with disease-related and overall survival.

**Results:**

Before and after matching for risk factors, recurrence was significantly more frequently diagnosed in OFTC patients during follow-up (17% vs. 8%; *p* value 0.037). Likewise, OFTC patients presented with a reduced mean disease-free survival of 17.9 years (95% CI 16.0–19.8) vs. 20.1 years (95% CI 19.0–21.1) in FTC patients (*p* value 0.027). Multivariate analysis revealed OFTC (HR 0.502; 95% CI 0.309–0.816) as the only independent prognostic factor for disease-free survival. Distant metastases of OFTC patients were significantly less iodine-avid (*p* value 0.014). Mean disease-specific and overall survival did not differ significantly (*p* value 0.671 and 0.687) during follow-up of median 6.9 years (3.7; 11.7 years).

**Conclusions:**

Our study suggests that recurrence is more often seen in OFTC patients. OFTC patients have a poorer prognosis for disease-free survival. Thus, OFTC and FTC behave differently and should be categorized separately. However, patients suffering from OFTC present with the same overall and disease-specific survival at the end of follow-up indifferent to FTC patients after initial RIT.

## Background

The incidence of thyroid cancer has increased globally [1]. Follicular thyroid carcinoma (FTC) is the second most common epithelial-derived thyroid cancer histotype after papillary carcinoma (PTC) [1, 2]. The incidence of FTC varies in different regions worldwide most likely depending on the iodine supply [3, 4]. Hürthle cell carcinoma (OFTC), also known as oxyphilic or oncocytic cell follicular thyroid carcinoma, represents about 3–5% of thyroid carcinomas [5–8]. Traditionally, OFTC has been considered as a variant of follicular thyroid cancer [9]. However, the latest thyroid tumor classification of the World Health Organization (WHO) of 2017 considered OFTC as an independent type of thyroid carcinoma and no longer as a subtype of FTC based on differences in clinicopathologic features and molecular alterations [8, 10]. Until now, there are controversial debates on almost all aspects of the disease including diagnosis, staging, treatment, and prognosis [11]. Some authors have reported a significantly more aggressive clinical course and a worse prognosis of both follicular and also papillary OFTC as compared with their non-oncocytic variants [7, 12–14]. In this context, it has been reported that only a minority of patients with distant metastasis showed positive I-131 radioactive iodine (RAI) uptake of these metastasis which contributes to a worse prognosis [15]. However, Jillaed et al. have demonstrated the association of improved survival in OFTC patients who were treated with radioiodine therapy (RIT) [16]. Recently, three studies have shown a similar prognosis of FTC and OFTC patients, especially if influencing risk factors are matched [11, 17, 18]. In most of the previously published studies, patients with different standard care procedures were included. In our department, all patients with OFTC and FTC have been treated with thyroidectomy followed by initial RIT on the basis of the same standard at that time of inclusion. Our goal was to determine patient characteristics and clinical long-term outcome in terms of disease-free, overall, and disease-specific survival, recurrence, and persistent disease in patients with OFTC in comparison with patients with FTC. Furthermore, we aimed to assess independent prognostic factors for disease-related and overall mortality.

## Methods

### Patients

Between 1993 and 2017, 3605 new patients with thyroid carcinoma were treated in our department. In this study, only patients diagnosed with oncocytic variant of follicular carcinoma (OFTC) and classical FTC with total or near total thyroidectomy followed by complementary initial radioiodine therapy were selected. Oncocytic variant of papillary thyroid carcinoma was suspended. Patients with a follow-up of less than 1 year in our center were also excluded. Finally, 378 patients aged over 18 years were enrolled in this study. Of these, 252 patients (67%) had been diagnosed histologically with classical FTC and 126 patients (33%) with OFTC. Data on demographic characteristics, tumor grade, persistent disease and recurrence during follow-up, and disease-free, disease-specific, and overall survival were collected. Pathology reports were reviewed for all patients to confirm the diagnosis of OFTC and FTC. Histopathology was adjusted to the 8th edition of the TNM classification of the American Joint Committee on Cancer (AJCC) in all patients [19, 20].

### Treatment and follow-up

All patients were treated with total or near total thyroidectomy. Additionally, lymph node dissection was performed in 37% of OFTC patients (*N* = 47) and in 42% of FTC patients (*N* = 106). Afterwards, patients were transferred consecutively to our clinic where RIT, RAI diagnostic whole-body scan, follow-up, and other staging examination were performed.

Initial RIT was administered orally either after thyroid hormone withdrawal when TSH level was above 30 μU/ml or after administration of 0.9 mg recombinant human thyrotropin alfa (rhTSH; Thyrogen®, Sanofi Genzyme) on two consecutive days with administration of radioiodine 24 h after the second injection. Between 1993 and November 2013, 3600 MBq I-131 (100 mCi) for patients with pT1/2 pN0 R0 c/pM0, 7400 MBq I-131 (200 mCi) for patients with pT3/4 and/or pN1 and/or R1, and 9200 MBq I-131 (250 mCi) for patients with known distant metastases (c/pM1) were administered for initial RIT. After November 2013 until end of study (2017), administered activity doses were 2000, 3700, 7400, and 9200 MBq I-131 (54, 100, 200, and 250 mCi) depending on patient age, TNMR-stage, and risk factors (e.g., Tg out of proportion, aggressive histology) based on the formerly ATA guidelines [21–23]. For additional courses of RIT, 7400 MBq I-131 (200 mCi) was given routinely. However, the final treatment activity could be modified within a certain range by the treating physician. Eventually, 3700–9200 MBq I-131 (100–250 mCi) was administered. After RIT, 2% of OFTC patients (*N* = 2) and 3% of FTC (*N* = 7) were treated with external radiation therapy (ERT) of the neck. During follow-up, serum TSH levels were maintained at levels < 0.1 μU/ml until 2006 in all patients. Afterwards, in all low-risk patients (T1/2 p/cN0/x cM0) and high-risk patients (pT3, p/cN1, or cM1 or pR1) presenting no evidence of disease (NED) for more than 5 years, TSH level was maintained at 0.2–0.4 μU/ml (2006–2009) and at 0.3–0.5 μU/ml (2009–2011) according to our clinical standard. After 2011, the TSH level was increased to 0.3–1.0 μU/ml in adaption to the ETA and ATA guidelines [24–26]. In all high-risk patients within the first 5 years and in all patients with evidence of disease (ED), TSH level was still maintained at levels < 0.1 μ/ml. In the 1st control during follow-up, a RAI diagnostic whole-body scan was performed in hypothyroidism or after rhTSH and TSH-stimulated serum thyroglobulin (Tg) level was measured. Follow-up examinations were usually performed every 3 months in the 1st year, every 6 months in the 2nd year after treatment, and annually thereafter. Additional imaging (chest X-ray, computed tomography (CT), magnetic resonance imaging, bone scintigraphy, or positron emission tomography/CT) was performed when medical physical examination or ultrasound was suspicious for recurrence or if Tg level was increased. Patients with ED during follow-up were treated based on a multidisciplinary tumor board decision including resection of local recurrence; resection of cervical lymph nodes; resection of bone metastases; atypical lung resection; ERT; chemotherapy; redifferentiation with rosiglitazone or isotretinoin; tyrosine kinase inhibitor (TKI) with lenvatinib, sorafenib, sunitinib, or pazopanib; and Rhenium-186-HEDP and DOTA-TATE therapies, respectively. Furthermore, 34% of these patients were treated with a ≥ 1 of these treatment options.

### Definition of endpoints

Patient outcomes were compared at four different time points: 1st control after RIT, during follow-up, last visit of follow-up, and end of study in analogy of a previously published study [27]: In the 1st control, patients were categorized into two groups, namely NED and ED. NED was assumed if Tg level was under the detection limit (1.6 ng/ml 1993–1996; < 1.0 ng/ml 1996–1997, < 0.5 ng/ml after 1998) while Tg recovery was in normal range. Additionally, RAI diagnostic whole-body scan had to be negative and the ultrasound examination had to show no local recurrence and/or lymphadenopathy. During follow-up, the rate of persistent disease and recurrence were evaluated. Persistent disease was supposed if stimulated or non-stimulated Tg level remained above the detection limit and if local recurrence, nodal, and distant metastases were not treated curatively. Recurrence was only evaluated in patients with initial M0/Mx status and complete response in the first follow-up examination including stimulated Tg under the detection limit and unsuspicious diagnostic RAI whole-body scan (*N* = 242). In these patients, recurrence was assumed if Tg-level increased above the detection limit or if tumor lesions were detected during follow-up. Disease-free survival was defined as the period from the first day of surgery to the first day of recurrence in M0/Mx patients. Furthermore, RAI uptake of distant metastases was assessed. Positive uptake was defined as increased uptake in all metastatic sites. Vice versa, insufficient uptake was defined as no uptake at all or only partial uptake of some metastases or initially positive uptake, but lack of uptake in additional cycles of RIT despite clinical ED. At the last follow-up visit, the rate of NED and ED was evaluated again. NED was assumed if Tg was under the detection rate and cervical sonography, and previous long-term follow-up of at least 1 year was inconspicuous. The end of our study included date and cause of death. Overall survival was defined as the period from surgery to death of any cause. Disease-specific survival was defined as the period from the first day of surgery to cancer-specific death. Additionally, patients were matched to exclude influence on survival in a subgroup analysis.

### Statistical analysis

Analysis was carried out with a statistical package for the social sciences software package (IBM SPSS, version 25.0, IBM North America). The quantitative variables were expressed as median (interquartile range, 25th and 75th percentiles), while the categorical ones were presented as numbers and percentages. To test normal distribution and comparison of variables between OFTC and FTC patients, the Kolmogorov-Smirnov test was applied. Comparisons of variables between patients with OFTC and FTC were performed using Student’s *t* test for parametric continuous data, the chi-quadrat test for ordinal data, Mann-Whitney *U* test for nonparametric continuous data, and Fisher exact test for categorical data. Statistical significance was defined as two-tailed *p* values < 0.05. Kaplan-Meier curves were displayed as mean (95%CI) as median survival was not reached. The multivariate regression model was applied to analyze prognostic factors associated with disease-related and overall survival. In the univariate analysis, gender, age, histology, TNMR stage, and tumor size have been evaluated based on previously published studies [11, 17]. In the multivariate analysis, only parameters that showed significant influence (*p* value < 0.05) on disease-specific or overall survival in the univariate analysis were included.

### Ethics statement

This retrospective study has been conducted in accordance with the ethical standards, according to the Declaration of Helsinki, and in accordance with national and international guidelines. The study was approved by the institutional ethics committee (# 18-768).

## Results

### Baseline characteristics

Patients’ baseline characteristics are presented in Tables 1 and 2. This retrospective study included a total of 378 patients. Thirty-three percent presented with OFTC (*N* = 126) and 67% with FTC (*N* = 252). Of note, at initial diagnosis, FTC patients presented significantly more often with M1-stage disease (4% in OFTC patients vs. 16% in FTC patients; *p* value < 0.001). Most of our patients (64% of OFTC patients and 55% of FTC patients) were treated with only one cycle of RIT. In 23% of OFTC patients and 21% of FTC patients, two cycles of RIT were administered. Of note, 20% of our patients (13% of OFTC patients and 24% of FTC patients) were treated with ≥ 3 cycles of RIT. Only FTC patients were treated with ≥ 7 cycles of RIT. Additional treatments are presented in Table 3. Overall, OFTC patients were followed up for median 7.4 years (3.7; 12.0 years) and FTC patients for 6.6 years (3.7; 11.6 years; *p* value 0.771).

**Table 1:** Patients’ baseline characteristics for OFTC and FTC

	Total *N* = 378	OFTC *N* = 126 (33%)	FTC *N* = 252 (67%)	*p* value
Age (years)	57.8 (47.0; 68.1)	57.8 (48.8; 67.6)	57.9 (46.5; 68.2)	0.75
Gender (female)	234 (62)	74 (59)	160 (64)	0.432
Time between surgery and RIT (months)	1.3 (1.1; 1.6)	1.3 (1.0; 1.5)	1.3 (1.2; 1.6)	0.33
Initial RAI dose (MBq)	3720 (3639; 3839)	3740 (3658; 3852)	3701 (3622; 3820)	0.713
> 1 cycle of RIT	159 (42)	46 (37)	113 (45)	0.075
Cumulative RAI dose (MBq)	3853 (3686; 11,585)	3864 (3700; 9978)	3849 (3679; 14,937)	0.967
Follow-up (years)	6.9 (3.7; 11.7)	7.4 (3.7; 12.0)	6.6 (3.7; 11.6)	0.771

Quantitative variables were expressed as median (interquartile range), while categorical ones were presented as numbers and percentages

*OFTC* Hürthle cell carcinoma, *FTC* follicular thyroid cancer, *SD* standard deviation

**Table 2:** Patients’ tumor characteristics and TNM classification for OFTC and FTC

		Total *N* = 378	OFTC *N* = 126 (33%)	FTC *N* = 252 (67%)	*p* value
Tumor size (cm)		3.2 (2.2; 4.5)	3.0 (2.1; 4.5)	3.2 (2.2; 4.4)	0.928
Tumor size	≤ 4 cm	235 (62)	80 (64)	155 (62)	0.922
	> 4 cm	92 (24)	30 (24)	62 (25)	
	Unknown	51 (14)	16 (13)	35 (14)	
pT	pT1a	11 (3)	2 (2)	9 (4)	0.904
	pT1b	61 (16)	23 (18)	38 (15)	
	pT2	159 (42)	50 (40)	109 (43)	
	pT3a	79 (21)	28 (22)	51 (20)	
	pT3b	7 (2)	3 (2)	4 (2)	
	pT4a	20 (5)	7 (6)	13 (5)	
	pT4b	5 (1)	1 (< 1)	4 (2)	
	pTx	36 (10)	12 (9)	24 (10)	
pN	pN0	136 (36)	42 (33)	94 (37)	0.669
	pN1a, pN1b, pN1	17 (5)	5 (4)	12 (5)	
	pNx	225 (60)	79 (63)	146 (58)	
cM	cM0, cMx	333 (88)	121 (96)	212 (84)	*0.001*
	pM1/cM1	45 (12)	5 (4)	40 (16)	
pR	pR0	262 (69)	85 (68)	177 (70)	0.896
	pR1	18 (5)	7 (6)	11 (4)	
	pR2	4 (1)	1 (< 1)	3 (1)	
	pRx	94 (25)	33 (26)	61 (24)	

Quantitative variables were expressed as median (minimum; maximum), while categorical ones were presented as numbers and percentages

*OFTC* Hürthle cell carcinoma, *FTC* follicular thyroid cancer. Significant values (*p*-value < 0.05) are italicized

**Table 3:** Multimodal treatment approach during follow-up

	OFTC *N* = 16	FTC *N* = 46	Total *N* = 62	*p* value
ERT:	6	17	24	1.0
Neck	3	3	6	
Brain	1	1	2	
Pleura	0	1	1	
Bone	2	13	15	
Surgical procedures:	10	17	27	0.089
Local recurrence	4	5	9	
Cervical lymph nodes	4	5	9	
Bone metastasis	2	6	8	
Atypical lung resection	3	2	5	
Chemotherapy	4	4	8	0.187
Rhenium-186-HEDP	0	1	1	1.0
DOTA-TATE therapy	0	2	2	1.0
Redifferentiation	1	9	10	0.43
TKI	4	7	11	0.452

Thirty-four percent of these patients were treated with ≥ 1 of these treatment options

*OFTC* Hürthle cell carcinoma, *FTC* follicular thyroid cancer, *ERT* external radiation therapy, *TKI* tyrosine kinase inhibitor

### Clinical outcome

**Fig. 1:**
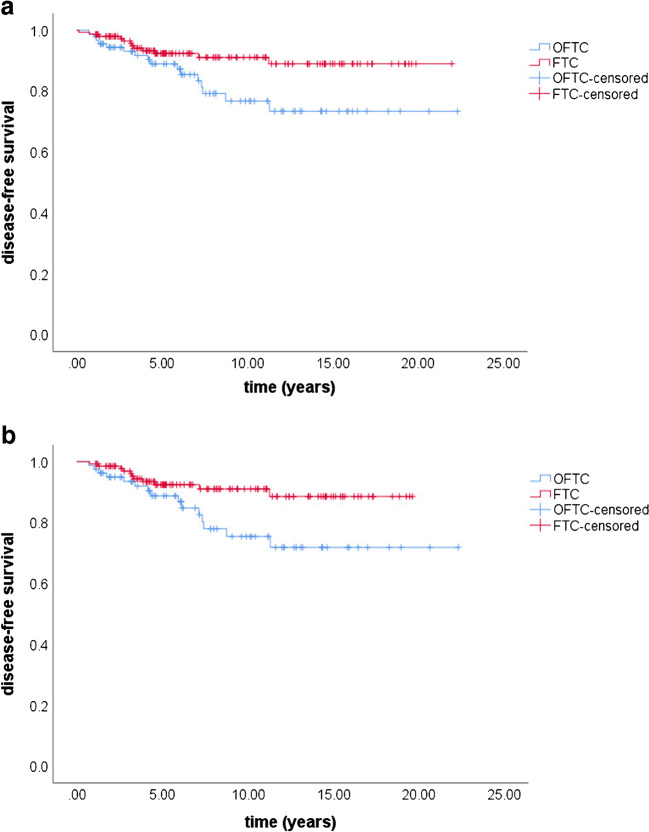
Disease-free survival in patients presenting NED in the 1st control before (**a**) and after matching (**b**). Before and after matching disease-free survival was significantly reduced in OFTC patients in comparison with FTC patients (before matching: *p* value 0.027, after matching: *p* value 0.024). OFTC: follicular Hürthle cell carcinoma; FTC: classical follicular thyroid carcinoma

In the first control after initial RIT, 27% of OFTC patients (*N* = 34/126) and 40% of FTC patients (*N* = 100/252) had still ED (*p* value 0.017). Of these, persistent disease was seen in 44% of OFTC patients (*N* = 15/34) and in 60% of FTC patients (*N* = 60/100), however without reaching statistical significance (*p* value 0.115). Apart, recurrence was significantly more often detected in OFTC patients (*N* = 16/92, 17%) than in FTC patients (*N* = 12/150, 8%; *p* value 0.037). Recurrence occurred after 3.2 years (1.4; 7.3 years) in the FTC group and after 4.2 years (1.6; 4.4 years) in the OFTC group (*p* value 0.41). Only 19% of recurrent OFTC patients (*N* = 3/16) and 17% of recurrent FTC patients (*N* = 2/12) were treated curatively at the end of follow-up (*p* value 0.887). Mean disease-free survival was 17.9 years (95% CI 16.0–19.8) in OFTC (*N* = 91, one patient missing) and 20.1 years (95% CI 19.0–21.1) in FTC patients (*N* = 150). Five-year, 10-year, and 20-year disease-free survival were 89%, 77%, and 73% in the OFTC and 92%, 91%, and 89% in the FTC group (*p* value 0.027; Fig. 1).

At the end of follow-up, 22% of OFTC patients (*N* = 28/126) and 29% of FTC patients (*N* = 72/252) had ED (*p* value 0.187). Complete remission (CR), partial remission (PR), stable disease (SD), and progression (PD) were seen in 78%, 4%, 5%, and 14% in OFTC patients and in 71%, 3%, 4%, and in 21% in FTC patients (*p* value 0.298).

Furthermore, at the end of our study, 19% of OFTC patients (*N* = 24/126) and 17% of FTC patients (*N* = 42/252) had died (*p* value 0.565). Thyroid carcinoma was the cause of death in 9% of OFTC patients (*N* = 11/126) and in 10% of FTC patients (*N* = 25/252; *p* value 0.314).

**Fig. 2:**
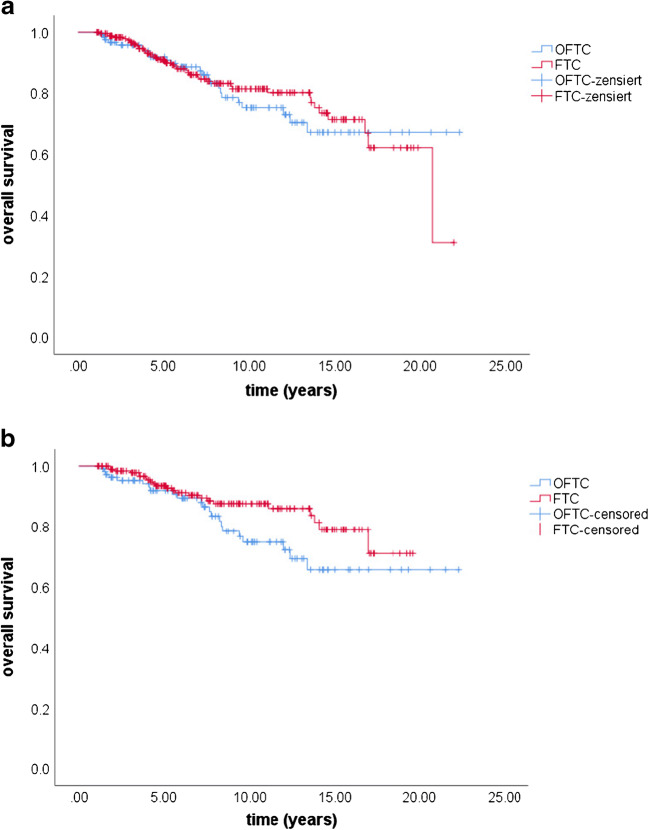
Overall survival in OFTC and FTC patients before (**a**) and after matching (**b**). Overall survival did not significantly differ between the two groups (before matching: *p* value 0.687, after matching: *p* value 0.075). OFTC: follicular Hürthle cell carcinoma; FTC: classical follicular thyroid carcinoma

**Fig. 3:**
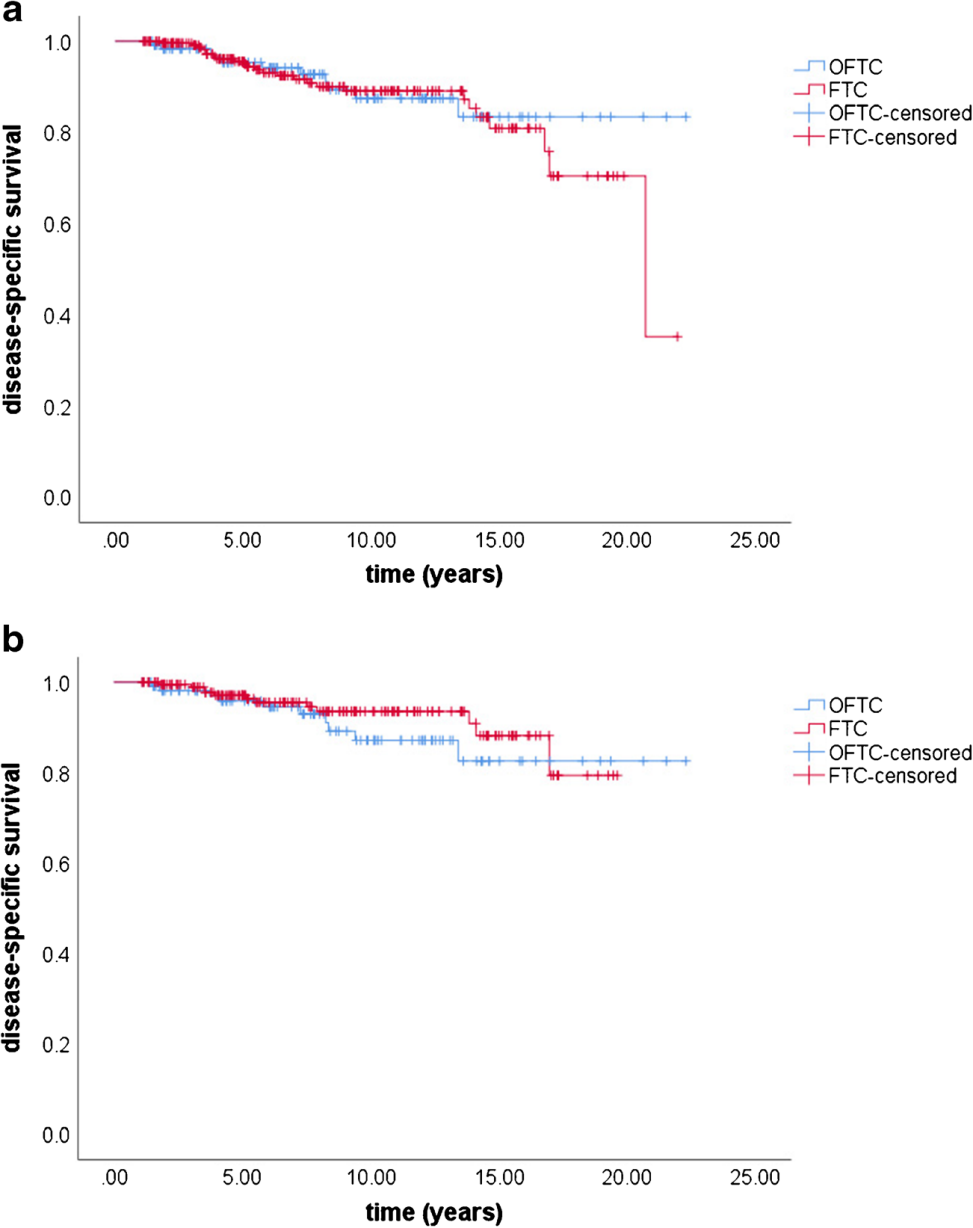
Disease-specific survival of OFTC and FTC patients before (**a**) and after matching (**b**). Likewise, no significant differences could be observed in disease-specific survival (before matching: *p* value 0.671, after matching: *p* value 0.351). OFTC: follicular Hürthle cell carcinoma; FTC: classical follicular thyroid carcinoma

Mean overall survival was 17.5 years (95% CI 15.9–19.2) in the OFTC group and 17.1 years (95% CI 15.9–18.4) in the FTC group. Five-year, 10-year, and 20-year overall survival were 92%, 75%, and 67% in the OFTC group and 91%, 81%, and 62% in the FTC group, respectively (*p* value 0.687). Mean disease-specific survival was 19.9 years (95% CI 8.6–21.3) in the OFTC group and 18.5 years (95% CI 17.3–19.8) in the FTC group. Five-year, 10-year, and 20-year disease-specific survival were 95%, 87%, and 84% in the OFTC group and 96%, 89%, and 70% in the FTC group, respectively (*p* value 0.671; Figs. 2 and 3).

### RAI uptake in distant metastases

A total of 2–16 cycles of RIT were performed to treat distant metastases in 14% of OFTC patients (*N* = 18) and in 25% of FTC patients (*N* = 62). In detail, there are 52 patients with pulmonary metastases (14 OFTC, 38 FTC), 35 with bone metastases (6 OFTC, 29 FTC), four with distant mediastinal and/or hilar metastases (4 FTC), four with liver metastases (1 OFTC, 3 FTC), two with soft tissue metastases (2 FTC), and one patient with brain metastases (FTC patient). In 17 patients, distant metastases involved different sites. Of note, in the OFTC group, only three patients (17%) presented with positive RAI uptake. On contrast, positive uptake was seen in 32 patients of the FTC group (52%). The difference in RAI uptake between the OFTC and FTC group was statistically significant (*p* value 0.014).

### Risk factors for disease-free, disease-specific, and overall survival

Tables 4, 5, and 6 reveal the results of regression analysis made to determine the factors associated with poorer patients’ prognosis for disease-free survival, disease-specific, and overall survival for all patients. OFTC is the only risk factor for disease-free survival in the multivariate analysis. The multivariate analysis for overall survival identified advanced age and T3/4 stages as significant risk factors. The results of multivariate regression analysis for disease-specific survival revealed that only advanced T3/4 stages were associated with poorer prognosis.

**Table 4:** Risk factors for disease-free survival

Covariate	Level	Univariate analysis		Multivariate analysis	
		HR (95% CI)	*p* value	HR (95% CI)	*p* value
Gender	Female				
	Male	1.188 (0.813–1.738)	0.374		
Age (years)	≤ 55 > 55	2.4 (1.085–5.31)	*0.031*	1.605(0.648–3.973)	0.307
Histology	FTC				
	OFTC	0.663 (0.456–0.965)	*0.032*	0.502 (0.309–0.816)	*0.005*
T-stage	T1/2				
	T3/4	3.493 (1.505–8.107)	*0.004*	4.4046 (0.5–32.713)	0.19
N-stage	N0/x				
	N1	6.389 (0.852–47.92)	0.071		
R-stage	R0/x				
	R1/2	5.221 (0.672–40.587)	0.114		
Tumor size	≤ 4 cm				
	> 4 cm	3.436 (1.535–7.688)	*0.003*	0.568 (0.067–4.849)	0.606

*OFTC* Hürthle cell carcinoma, *FTC* follicular thyroid cancer, *T* tumor, *N* nodus, *M* metastasis, *R* resection, *HR* hazard ratio, *CI* confidence interval. Significant values (*p*-value < 0.05) are italicized

**Table 5:** Risk factors for overall mortality

Covariate	Level	Univariate analysis		Multivariate analysis	
		HR (95% CI)	*p* value	HR (95% CI)	*p* value
Gender	Female				
	Male	1.318 (1.032–1.683)	*0.027*	1.457 (0.956–2.223)	0.08
Age (years)	≤ 55				
	> 55	8.614 (3.712–19.99)	*< 0.001*	22.608 (2.681–190.657)	*0.004*
Histology	OFTC				
	FTC	0.95 (0.739–1.221)	0.687	0.724 (0.424–1.236)	0.236
T-stage	T1/2				
	T3/4	5.217 (2.91–9.356)	*< 0.001*	5.024 (1.267–19.924)	*0.022*
N-stage	N0/x				
	N1	3.057 (1.388–6.731)	*0.006*	4.621 (0.762–28.028)	0.096
M-stage	M0/x				
	M1	4.529 (2.708–7.575)	*< 0.001*	2.103 (0.798–5.541)	0.133
R-stage	R0/x				
	R1/2	7.463 (3.552–15.68)	*< 0.001*	2.727 (0.949–7.838)	0.062
Tumor size	≤ 4 cm				
	> 4 cm	2.661 (1.48–4.785)	*0.001*	0.386 (0.104–1.436)	0.155

*OFTC* Hürthle cell carcinoma, *FTC* follicular thyroid cancer, *T* tumor, *N* nodus, *M* metastasis, *R* resection, *HR* hazard ratio, *CI* confidence interval. Significant values (*p*-value < 0.05) are italicized

**Table 6:** Risk factors for disease-specific mortality

Covariate	Level	Univariate analysis		Multivariate analysis	
		HR (95% CI)	*p* value	HR (95% CI)	*p* value
Gender	Female				
	Male	1.286 (0.92–1.798)	0.141		
Age (years)	≤ 55				
	> 55	7.106 (2.498–20.211)	*< 0.001*	113,294.32 (0–>1000)	0.926
Histology	OFTC				
	FTC	1.08 (0.757–1.541)	0.671		
T-stage	T1/2				
	T3/4	14.92 (5.124–43.441)	*< 0.001*	33.255 (2.857–387100)	*0.005*
N-stage	N0/x				
	N1	4.286 (1.644–11.174)	*0.003*	4.546 (0.274–75.38)	0.291
M-stage	M0/x				
	M1	5.835 (2.97–11.462)	*< 0.001*	2.103 (0.798–5.541)	0.231
R-stage	R0/x				
	R1/2	8.88(3.312–23.807)	*< 0.001*	2.077 (0.629–6.866)	0.062
Tumor size	≤ 4 cm				
	> 4 cm	5.816 (2.46–13.747)	*< 0.001*	0.535 (0.111–2.575)	0.435

*OFTC* Hürthle cell carcinoma, *FTC* follicular thyroid cancer, *T* tumor, *N* nodus, *M* metastasis, *R* resection, *HR* hazard ratio, *CI* confidence interval. Significant values (*p*-value < 0.05) are italicized

### Matching for patients’ characteristics—subgroup analysis

Presenting with significant differences in cM1-stage, OFTC and FTC patients were matched to exclude influence on survival in a subgroup analysis. Patients were matched by age, sex, and TNM classification, R-stage, tumor size, and RAI dose.

There were 310 patients (111 OFTC, 199 FTC) included in the matched patient group analysis. After matching, 27% of the OFTC group and 31% of the FTC had evidence of disease after the 1st RIT (*p* value 0.517). In patients who presented with ED in the 1st control, persistent disease was found in 43% of OFTC and in 44% of FTC patients (*p* value 1.0). Recurrence during follow-up was detected significantly more often in OFTC patients than in FTC patients (19% vs. 8%; *p* value 0.03). Mean disease-free survival was 17.7 years (95% CI 15.6–19.7) in the OFTC group (*N* = 81) and 18.0 years (95% CI 17.0–18.9) in the FTC group (*N* = 137). The 5-year, 10-year, and 20-year disease-free survival were 89%, 75%, and 72% in patients with OFTC and 93%, 91%, and 89% in patients with FTC, respectively (*p* value 0.024).

At the last follow-up visit, 23% of the OFTC group and 18% of the FTC had ED (*p* value 0.373). CR, PR, SD, and PD were seen in 78%, 5%, 4%, and 14% in OFTC patients and in 82%, 2%, 4%, and 13% in FTC patients (*p* value 0.412). At the end of our study, overall mortality rate was 20% of OFTC and 12% of FTC patients (*p* value 0.063). Mean overall survival was 17.3 years (95% CI 15.6–19.1) in the OFTC group and 17.1 years (95% CI 16.1–18.2) in the FTC group. The 5-year, 10-year, and 20-year overall survival rate were 92%, 75%, and 66% in patients with OFTC and 94%, 88%, and 71% in patients with FTC respectively (*p* value 0.075).

Disease-specific mortality rate was 9% in the OFTC and 6% in the FTC group (*p* value 0.768). Mean disease-specific survival was 19.8 years (95% CI 18.4–21.3) in the OFTC group and 18.1 years (95% CI 17.3–19.0) in the FTC group. The 5-year, 10-year, and 20-year disease-specific survival rate were 96%, 87%, and 83% in patients with OFTC and 97%, 94%, and 79% in patients with FTC, respectively (*p* value 0.351).

## Discussion

Little is known about the aggressiveness of OFTC in comparison with classical FTC, because OFTC is a relatively rare disease and less common than other well-differentiated thyroid carcinoma. The limited clinical experience and the small number of available investigational studies make it difficult to recommend appropriate clinical management and follow-up strategies. The clinical outcome of OFTC is still discussed controversially. In fact, four different US studies which searched for the same outcome and were performed on the same Surveillance, Epidemiology, and End Results (SEER) database came to different results [5, 6, 28, 29]. Thus, the prognosis of OFTC in comparison with classical FTC still needs to be studied. We aimed to assess the differences in patients’ characteristics and the clinical outcome in patients with OFTC compared with patients with FTC. Of note, our study consists of a large patient cohort following thyroidectomy and contemporary initial RIT.

In our study, OFTC patients presented less often with distant metastases (4% vs. 16%, *p* value 0.001). Likewise, in another study, distant metastasis at presentation was significantly less often diagnosed in OFTC patients than in FTC patients (4% vs. 13%; *p* value 0.03) [17]. This is in contrast to a previously published study in which OFTC patients tended more likely to present with distant metastases (8% vs. 3%, *p* value 0.078) [18]. Patients presenting with risk factors such as advanced tumor stages might have been more likely transferred to a tertiary care center which has the permission to use higher radiation doses and explaining our relevant number of cM1 patients. However, the relevant and higher number of FTC patients presenting with cM1 remains unclear.

We observed that recurrences were more frequently diagnosed in OFTC patients than in FTC patients (17% vs. 8%, *p* value 0.037), indicating that the oncocytic variant has a more aggressive course. These findings are in line with a previously published study including a smaller patient group of 33 OFTC and 85 FTC patients who were treated by lobectomy, total or less than the total thyroidectomy followed by RIT in almost half of the patients [13]. The authors observed that more OFTC patients developed recurrent disease (24% vs. 8%, *p* value 0.05) [13]. In a similar setting, persistence/recurrence occurred in OFTC patients also more frequently (13% in the OFTC and 4% in the FTC group, *p* value 0.011) [18]. In general, recurrence ranges from 11 to 43% in OFTC patients [6, 30–32]. Thus, the recurrence rate in OFTC patient is relevant. Furthermore, recurrence was detected almost 1 year earlier in FTC patients than in OFTC patient, although statistically not significant. It has been described in a previously published study that nodules of OFTC were located not in true lymph nodes but in soft tissues of the neck unassociated with lymphoid tissue. Furthermore, if lymph node metastases were present, they were often microscopic (< 5 mm) [33]. Furthermore, the lack of RAI uptake in distant metastases might have also resulted in a delayed detection of recurrence in OFTC patients. Thus, diagnosis of these metastases presents additional challenges [8] which might explain why recurrence in OFTC patients was detected at a later time point. In addition, we noticed a poorer disease-free survival in OFTC patients. In fact, multivariate analysis revealed OFTC as the only independent prognostic factor for disease-free survival.

All our patients were treated with total or near total thyroidectomy followed by initial RIT. In previously published studies, the percentage of thyroidectomy ranged from 27 to 100% [6, 11, 13, 17, 18, 28, 30–32, 34]. Surgery for thyroid carcinoma is an important component of a multimodal treatment approach. Former guidelines have suggested total thyroidectomy as the primary surgical treatment option for nearly all thyroid carcinoma greater than 1 cm with or without evidence of loco-regional or distant metastases [35]. The benefit of consecutive RIT in OFTC patients is still not clarified sufficiently. Certainly, ablative RIT of residual thyroid tissue can improve early detection of recurrences based on Tg measurement [24]. However, it is claimed that OFTC patients demonstrate a poorer iodine avidity and are therefore less responsive to RIT [36]. Indeed, in a previously published study, most of the known metastases did not show RAI uptake and furthermore not all metastatic sites showed uniform RAI uptake [15]. In contrast, Besic et al. found radioiodine uptake in recurrent disease or in distant metastases in 11 of 16 OFTC patients [37]. Thus, the efficacy of RIT in metastatic OFTC patients is still controversy. In our study, distant metastases of OFTC patients were significantly less iodine-avid. Indeed, only 11% of metastatic OFTC patients have shown RAI uptake which is in line with a previously published study. In this study, only 10% of OFTC patients with bone and pulmonary metastases demonstrated RAI uptake [15]. Nevertheless, Jillard et al. have shown that survival was significantly improved in OFTC patients (pT1 with N1 or M1, and pT2–4 with any N or M) who received RIT in comparison with OFTC patients without RIT [16]. Furthermore, it has been shown in a subgroup analysis that OFTC patients who received RIT for adjuvant ablation therapy confer better outcomes compared either with patients who did not receive RIT or with patients who received RIT for residual disease [15]. Thus, in our department, RIT is performed in metastatic OFTC patients. However, if the use of RIT is limited by missing RAI uptake, other therapeutic options for these patients should be discussed in a multidisciplinary tumor board.

Our intention was much more to analyze if OFTC patients have a poorer outcome than FTC patients due to a more aggressive histology or to different risk factors. Therefore, we performed a Cox regression analysis to determine risk factors which were associated with poorer disease-specific and overall survival. Independent prognostic factors for disease-specific survival are described inconsistently. Male gender, advanced age of patients, advanced TNM (tumor, nodes, metastasis) stages, and residual tumor after thyroidectomy have been noted to influence outcome negatively [11, 13, 18, 29, 30, 34, 38–40]. In our study, we could also identify these risk factors in the univariate analysis. Interestingly, tumor size of > 4 cm was a risk factor for overall and disease-specific survival in the univariate but not in the multivariate analysis. Indeed, it is still debatable if tumor size represents a risk factor at all [30, 32]. In our study, T-stage was the only risk factor that was identified in the multivariate analysis. Besides tumor size, gross extrathyroidal extension invading strap muscles or into major neck structures is part of the definition of T. Indeed, extrathyroidal extension was identified as a risk factor by others [15, 34, 38]. Thus, T-stage including gross extrathyroidal extension may be even more associated with poorer survival than the tumor size itself. However, the histological variant (OFTC vs. FTC) was not a risk factor for overall and disease-specific survival per se. At the end of our study, patients suffering from OFTC presented with the same overall and disease-specific survival at the end of follow-up indifferent to FTC patients after a follow-up time of almost 7 years.

### Limitations

Firstly, the main limitation certainly represents the retrospective design; also, this study is from a single center. Histopathological data had to be adjusted to the 8th edition of UICC in order to compare TNM status. As a consequence, in some patients, TNM classification could not be adapted. Furthermore, at the time of inclusion, SPECT or even hybrid imaging was not available and consequently reliable differentiation between thyroid remnant tissue and radioiodine-avid lymph node metastases was not possible. Furthermore, at the time of inclusion, Tg assays were not as sensitive as today which might have hampered early detection of recurrence. Furthermore, the treatment regimens varied over time ranging from a quite aggressive approach up to a more personalized therapy which takes various risk factors into account. However, this shift applies to both groups equally. We focused on the long-term outcome, and therefore, we had to include these patients. Indeed, we still believe that our results can be transferred to patients nowadays. Last, the median follow-up time of almost 7 years might be too short to observe differences in overall and disease-specific survival. The good overall prognosis of differentiated FTC and OFTC may require a large patient cohort and an extreme long-term follow-up to show differences in survival.

## Conclusion

Our study suggests that recurrence is more often seen in OFTC patients. Furthermore, OFTC patients have a poorer patients’ prognosis for disease-free survival. However, OFTC patients present with the same overall and disease-specific survival indifferent to FTC patients after initial RIT.

## Data Availability

The datasets generated during and/or analyzed during the current study are available from the corresponding author or first author on reasonable request.
